# A case of minor *BCR-ABL1* positive acute lymphoblastic leukemia following essential thrombocythemia and originating from a clone distinct from that harboring the *JAK2*-V617F mutation

**DOI:** 10.1186/2162-3619-3-6

**Published:** 2014-02-17

**Authors:** Yuya Nagai, Masahiro Kawahara, Noriko Sugino, Yayoi Shimazu, Masakatsu Hishizawa, Kouhei Yamashita, Norimitsu Kadowaki, Akifumi Takaori-Kondo

**Affiliations:** 1 Department of Hematology and Oncology, Graduate School of Medicine, Kyoto University, 54 Shogoin-Kawahara-cho, Sakyo-ku, Kyoto 606-8507, Japan

**Keywords:** *JAK2*-V617F, Myeloproliferative neoplasms, BCR-ABL1, Lymphoblastic leukemia, Tyrosine kinase inhibitor, Resistant clone

## Abstract

Here we report on a case of Philadelphia chromosome positive B lymphoblastic leukemia (Ph^+^ALL), which developed following a long duration of essential thrombocythemia (ET). A mutational analysis of *Janus Kinase 2 (JAK2)* revealed that the V617F mutation was present in granulocytes and in hematopoietic stem and progenitor cells (HSPCs), but not in the CD34^+^CD19^+^ population that mostly consists of Ph^+^ALL cells, indicating that this Ph^+^ALL clone did not originate from the ET clone carrying the *JAK2*-V617F mutation. The minor *BCR-ABL1* fusion was detected not only in the CD34^+^CD19^+^ population but also in HSPCs and granulocytes, indicating that the Philadelphia chromosome was acquired in an early hematopoietic stage at least prior to the commitment to B cell development. Upon dasatinib treatment, the minor *BCR-ABL1* transcript rapidly disappeared in HSPCs but persisted in the CD34^+^CD19^+^ population. A relapse of Ph^+^ALL occurred nine months later without the disappearance of the minor *BCR-ABL1* transcript in the bone marrow cells during the treatment course, suggesting that a resistant Ph^+^ALL clone may have arisen or been selected in the committed B cells rather than in HSPCs. This case report may partly contribute to filling the gap between previous data acquired from mice experiments and the phenomenon in real patients.

## Background

Myeloproliferative neoplasms (MPNs) are a group of stem cell disorders including polycythemia vera (PV), essential thrombocythemia (ET), and primary myelofibrosis (PMF), all of which are characterized by the overproduction of mature blood cells. It is well known that patients with MPNs often develop acute myeloid leukemia (AML) [1], but also it has been occasionally reported that MPNs may be associated with lymphoid malignancies including non-Hodgkin lymphoma, chronic lymphocytic leukemia (CLL), and multiple myeloma [2,3]. However, a genetic association between B lymphoblastic leukemia (B-ALL) and MPNs is rarely observed [4,5].

The *JAK2*-V617F mutation is one of the major causes of MPNs and is present in the vast majority of these patients (90–95% of PV patients and 50–60% of ET and PMF patients) [6]. Intriguingly, transformation of the *JAK2*-V617F positive clones to AML is observed mainly in cases of primary or secondary myelofibrosis while AML clones arising directly from PV and ET are mostly *JAK2* wild-type, indicating clonal heterogeneity of MPNs [1]. Similarly, in cases of CLL or diffuse large B cell lymphoma following MPNs, the *JAK2-*V617F mutation is detected either in both MPN cells and B lymphoid tumor cells or in only MPN cells [3]. *JAK2* mutations at other residues, such as R683, are also observed in high-risk childhood acute lymphoblastic leukemia [7], supporting the theory that *JAK2* mutations may confer a growth advantage on B lymphocytes.

The *BCR-ABL1* fusion kinase encoded by the Philadelphia (Ph) chromosome, which arises from the chromosomal translocation t(9;22), is a major cause of chronic myeloid leukemia (CML) as well as of Ph^+^ acute lymphoblastic leukemia (Ph^+^ALL). CML is presumed to arise from aberrant Ph^+^ stem cells which are enriched in the CD34^+^CD38^-^ hematopoietic stem cell population. Tyrosine kinase inhibitors (TKIs) such as imatinib and dasatinib that specifically target the BCR-ABL1 kinase have improved the outcomes of patients with CML but have failed to provide a cure for the disease. The maintenance of leukemia stem cells (LSCs), which are capable of engraftment in immunodeficient mice, does not require the BCR-ABL1 kinase activity [8], and hence the disease usually relapses once TKI treatment is discontinued [9].

Ph^+^ALL is a subtype of B-ALL with a particularly poor prognosis even in the current era of TKIs. It remains unclear whether Ph^+^ALL arises from the CD34^+^CD19^-^ population before the commitment to B cell development [10] or from the CD34^+^CD19^+^ pro-B population [11]. LSCs of B-ALL do not appear to be enriched in a specific population in xenotransplanted mice [12,13] but it is unclear whether a specific population resistant to TKIs, as in the case of CML-LSCs, exists in Ph^+^ALL patients.

In this case report, we describe a particular case of Ph^+^ALL that followed ET, examine which cell population is the target for two major genetic alterations, *JAK2*-V617F and minor *BCR-ABL1* to understand the clonal architecture between Ph^+^ALL and ET, and investigate whether the sensitivity of subpopulations of Ph^+^ALL to dasatinib differs.

## Case presentation

In 1995, a 51-year-old woman was diagnosed as ET with the clinical examinations revealing a platelet count of 1240 × 10^9^ /L, total white blood cell count of 7.9 × 10^9^ /L, hemoglobin levels of 12.1 g/dl, and a marked proliferation of large, mature megakaryocytes in the bone marrow aspirate. She had been treated with only an anti-thrombotic agent for more than ten years except for one year with a cytoreductive therapy utilizing hydroxyurea that was discontinued in 2003 due to intolerance, and her disease had been well controlled without any thrombotic events or any signs of progression to terminal myelofibrosis. In October 2011, at the age of 67, the platelet count suddenly decreased to 336 × 10^9^ /L and blasts were detected with a total leukocyte count of 8.9 × 10^9^ /L (14% blasts) in the peripheral blood. Computed tomography (CT) scans of the abdomen and pelvis showed no splenomegaly. A bone marrow examination revealed hypercellularity with increased numbers of megakaryocytes and leukemic blasts, accounting for 76% of the total nucleated cells. Fluorescence-activated cell sorting (FACS) analysis showed a B-ALL phenotype (CD34^+^ CD19^+^ CD10^+^ CD13^+^ HLA-DR^+^) and Southern blot analysis clearly demonstrated monoclonality with a rearrangement of the immunoglobulin heavy chain gene. Cytogenetic analysis as well as Fluorescence in situ hybridization (FISH) analysis revealed clonal abnormalities with translocation t(9;22)(q34; q11.2) of the Ph chromosome and monosomy 7. The presence of the minor *BCR-ABL1* fusion transcript was confirmed using a reverse transcription–polymerase chain reaction (RT-PCR) and direct Sanger sequencing. Based on these results, we diagnosed Ph^+^ALL that may have transformed from ET.

**Figure 1:**
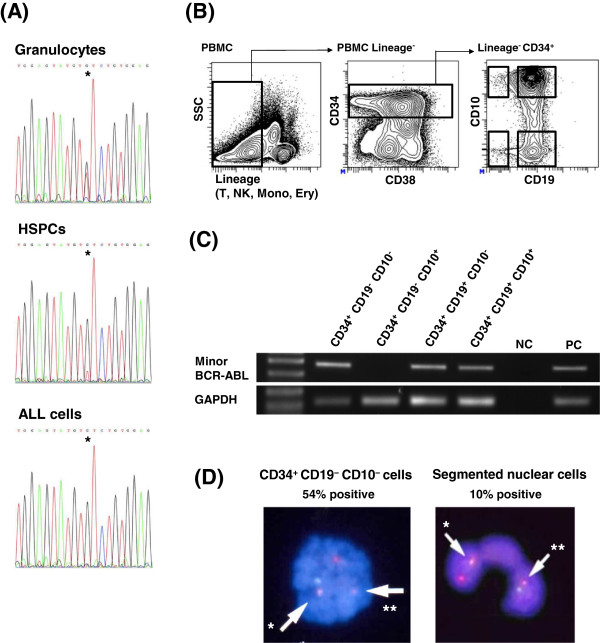
**Analysis of the molecular based clonal architecture. (A)** Sequencing of *JAK2*. Granulocytes and FACS-sorted lineage^-^CD34^+^ cells (HSPCs) and CD34^+^CD19^+^ B-ALL cells were analyzed. The *JAK2*-V617F mutation was not detected in B-ALL cells. Asterisk indicates nucleotide 1849 of *JAK2*. Lineage markers included CD2, CD3, CD4, CD7, CD8, CD10, CD11b, CD14, CD19, CD20, CD56 and CD235. **(B)** FACS analysis and sorting of PBMCs at diagnosis. The gating strategy to isolate four populations is shown. Lineage markers included CD2, CD3, CD4, CD7, CD8, CD11b, CD14, CD56 and CD235. **(C)** RT-PCR analysis for each population (gated in (B)). Plasmids containing the amplified region of minor *BCR-ABL* or *GAPDH* were used as positive controls (PC). Distilled water was used as the negative control (NC). The left lane shows the size marker. The Minor *BCR-ABL* transcript was also detected in CD34^+^CD19^-^CD10^-^ cells. **(D)** FISH analysis of *BCR-ABL* utilizing probes of Vysis LSI ASS-ABL for 9q34 (red) and Vysis LSI BCR for 22q11.2 (green). One red-green fusion signal specified by the arrow* indicates the presence of *BCR-ABL*. One smaller red signal specified by the arrow** indicates the remaining part of 9q34. Translocation t(9;22) was detected in both CD34^+^CD19^-^CD10^-^ cells at diagnosis and in segmented nuclear cells four days after the initiation of dasatinib treatment.

Subsequently, after acquiring a written informed consent, we investigated the *JAK2*-V617F mutational status in peripheral granulocytes isolated by Percoll density gradient centrifugation and in FACS-sorted lineage^-^CD34^+^ HSPCs and CD34^+^CD19^+^ B-ALL cells from peripheral blood mononuclear cells (PBMCs) at diagnosis. The sequencing analysis performed as previously described [14] showed that the *JAK2*-V617F mutation was present clearly in granulocytes and to a lesser extent in HSPCs, but not at all in B-ALL cells (Figure 1A). These results indicate that the B-ALL clone did not originate from the ET clone with the *JAK2*-V617F mutation.

Next in order to determine the stage in which the Ph chromosome was initially acquired, we separated CD34^+^ cells into four populations according to CD10 and CD19 expression (Figure 1B) after the exclusion of populations with lineage markers other than B cell markers including CD10, CD19, and CD20, and then performed the RT-PCR for the minor *BCR-ABL1* transcript. As expected, the amplification of the transcript was observed in the CD34^+^CD19^+^CD10^+^ and the CD34^+^CD19^+^CD10^-^ populations both of which are committed to B cell development. However, the CD34^+^CD19^-^CD10^-^ population which enriches HSPCs also expressed the minor *BCR-ABL1* transcript (Figure 1C). FISH analysis revealed that 54% of CD34^+^CD19^-^CD10^-^ cells as well as 10% of granulocytes that are defined as segmented nuclear cells carried the Ph chromosome (Figure 1D). Taken together, these findings suggest that the Ph chromosome was acquired during an early hematopoietic stage before the commitment to B cell development.

**Figure 2:**
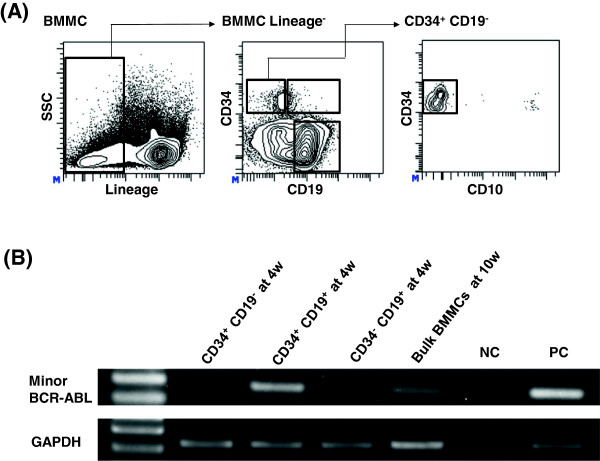
**Chase of the minor BCR-ABL1 positive clone during clinical course. (A)** FACS analysis and sorting of BMMCs at four weeks after the initiation of dasatinib treatment. The gating strategy to isolate three populations is shown. Lineage markers include CD2, CD3, CD4, CD7, CD8, CD11b, CD14, CD56 and CD235. **(B)** RT-PCR analysis for each population (gated in (A)) at four weeks and bulk BMMCs at ten weeks. Minor *BCR-ABL* transcripts was clearly detected only in CD34^+^CD19^+^ cells but not in CD34^+^CD19^-^ at four weeks and still detected in bulk BMMCs in low levels at ten weeks. Positive control (PC), plasmids containing the amplified region of minor *BCR-ABL* or the *GAPDH* gene; negative control (NC), distilled water.

After the diagnosis, the patient was treated with dasatinib and prednisolone. Four weeks later, we observed a great reduction of leukemia cells. To investigate whether the most primitive population in Ph^+^ALL were less sensitive to TKIs as in the case of CML, we separated bone marrow mononuclear cells (BMMCs) into three populations according to the expression level of CD34 and CD19 (Figure 2A) and performed RT-PCR for minor *BCR-ABL1*. Contrary to our expectation, the minor *BCR-ABL1* transcript was no longer detected in the CD34^+^CD19^-^ population that was almost negative for CD10 corresponding to the CD34^+^CD19^-^CD10^-^ population depicted in Figure 1B-D, which carried the minor *BCR-ABL1* transcript at diagnosis. However, the transcript was still detected in the CD34^+^CD19^+^ population (Figure 2B). Ten weeks later, the patient achieved cytogenetic remission with an increase in the platelet count, suggesting that the ET clone had repopulated during treatment. However, the minor *BCR-ABL1* transcript was still detected at low levels in the bulk bone marrow cells (Figure 2B), and finally the Ph^+^ALL relapsed with the T315I mutation nine months later, indicating that a resistant clone may not always derive from the most primitive population such as HSPCs.

## Conclusions

Cases of ET and B-ALL comorbidity are very rare. We initially thought this case was a transformation of ET to B-ALL similar to lymphoid crisis of CML, but were proven wrong when the mutational analysis of *JAK2* clearly showed that the B-ALL clone did not originate from the ET clone with the *JAK2*-V617F mutation. These results raise two hypotheses. One is that a microenvironment generated by MPNs may contribute to the development of an aberrant clone. A recent report that MPNs can remodel the bone marrow niche may support this hypothesis [15]. The other is that an aberrant clone may develop independently to ET or B-ALL with the additional hit of the *JAK2*-V617F mutation or the translocation t(9;22) respectively. A previous report that del (11q) was detected both in a *JAK2*-V617F positive MPN clone and in a *JAK2*-V617F negative AML clone in the same patient may support this hypothesis [16]. However, Monosomy 7 which was positive at diagnosis of Ph^+^ALL was not detected in bone marrow cells in cytogenetic remission after dasatinib treatment, indicating it was probably a second hit after the translocation t(9;22) in Ph^+^ALL cells. Since mutations in epigenetic regulators are common in MPNs [17], we also performed the mutational analysis for several genes such as the terminal exon of *DNMT3A* including R882, exon 4 of *IDH1* including R132, exon 4 of *IDH2* including R140 and R172, and exon 3 to 11 of *TET2*, but failed to find any founder mutations. The recent progress of high-throughput sequencing may resolve this question in the future.

LSCs of CML are enriched in the CD34^+^CD38^-^ hematopoietic stem cell population while those of B-ALL are not enriched in a specific population as several reports demonstrated that various phenotypically separated populations such as HSPCs and pre-B cells possess the engraftment capacity in immunodeficient mice [12,13]. Given that LSCs of CML are resistant to TKIs due to their quiescence and independent maintenance from BCR-ABL kinase [8], LSCs of B-ALL might be defined as a population that is not eliminated by chemotherapy and TKI treatment in patients. However, they have not yet been well studied in real Ph^+^ALL patients treated with TKIs. The LSC-like population of Ph^+^ALL in this case might exist in the CD34^+^CD19^+^ B cell committed population rather than in lineage^-^CD34^+^ HSPCs, since the *BCR-ABL1* transcript remained in the former but quickly disappeared in the latter at four weeks after therapy initiation, despite the latter being more primitive. Although we could not analyze whether the T315I mutation was acquired or selected in this committed population due to the limited sample size, it would be of value to examine similar cases in more detail throughout the clinical courses, in order to fill the current knowledge gap between results from mice experiments and findings from real patients and to be able to eradicate residual leukemic clones in such patients.

### Consent

Written informed consent was obtained from the patient for publication of this Case report and any accompanying images. A copy of the written consent is available for review by the Editor-in-Chief of this journal.

## Abbreviations

ALL: Lymphoblastic leukemia; ET: Essential thrombocythemia; JAK2: Janus Kinase 2; HSPCs: Hematopoietic stem and progenitor cells; MPNs: Myeloproliferative neoplasms; PV: Polycythemia vera; PMF: Primary myelofibrosis; AML: Acute myeloid leukemia; CLL: Chronic lymphocytic leukemia; CML: Chronic myeloid leukemia; TKIs: Tyrosine kinase inhibitors; LSCs: Leukemia stem cells; CT: Computed tomography; FACS: Fluorescence-activated cell sorting; FISH: Fluorescence in situ hybridization; RT-PCR: Reverse transcription–polymerase chain reaction; PBMCs: Peripheral blood mononuclear cells; BMMCs: Bone marrow mononuclear cells.

## Competing interests

The authors declare that they have no relevant financial interests.

## Authors’ contributions

YN performed all experiments and wrote the manuscript. MK designed the study and all experiments, and wrote the manuscript. KY cared for the patient. NS and YS performed a part of experiments. MH, NK and AT helped to draft the manuscript. All authors read and approved the final manuscript.
